# Decoding genome-wide GadEWX-transcriptional regulatory networks reveals multifaceted cellular responses to acid stress in *Escherichia coli*

**DOI:** 10.1038/ncomms8970

**Published:** 2015-08-10

**Authors:** Sang Woo Seo, Donghyuk Kim, Edward J. O'Brien, Richard Szubin, Bernhard O. Palsson

**Affiliations:** 1 Department of Bioengineering, University of California San Diego, La Jolla, California 92093, USA; 2 Department of Pediatrics, University of California San Diego, La Jolla, California 92093, USA; 3 Novo Nordisk Foundation Center for Biosustainability, Technical University of Denmark, 2800 Lyngby, Denmark

## Abstract

The regulators GadE, GadW and GadX (which we refer to as GadEWX) play a critical role in the transcriptional regulation of the glutamate-dependent acid resistance (GDAR) system in *Escherichia coli* K-12 MG1655. However, the genome-wide regulatory role of GadEWX is still unknown. Here we comprehensively reconstruct the genome-wide GadEWX transcriptional regulatory network and RpoS involvement in *E. coli* K-12 MG1655 under acidic stress. Integrative data analysis reveals that GadEWX regulons consist of 45 genes in 31 transcription units and 28 of these genes were associated with RpoS-binding sites. We demonstrate that GadEWX directly and coherently regulate several proton-generating/consuming enzymes with pairs of negative-feedback loops for pH homeostasis. In addition, GadEWX regulate genes with assorted functions, including molecular chaperones, acid resistance, stress response and other regulatory activities. These results show how GadEWX simultaneously coordinate many cellular processes to produce the overall response of *E. coli* to acid stress.

## Abstract (web summary)

GadEWX regulons play a critical role in transcription regulation in response to acid stress. By reconstructing genome-wide GadEWX transcriptional network, here the authors show how GadEWX simultaneously coordinates many other cellular processes to produce the overall response of *E. coli* to acid stress.

## Graphical abstract

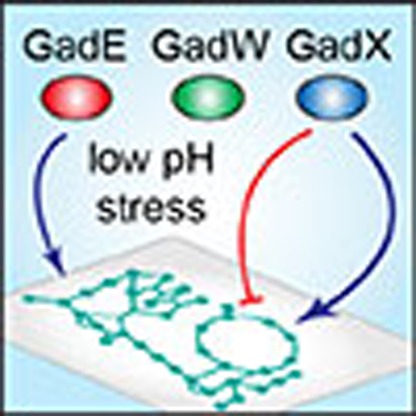

Bacteria depend on maintaining pH homeostasis, as most proteins have optimal ranges of pH where they function^1^. Diverse strategies for pH sensing and homeostasis allow bacteria to tolerate or grow at acidic external pH values. One of the most well-studied model organisms, *Escherichia coli* K-12 MG1655, has sophisticated acid resistance (AR) systems to survive in acidic pH environments. The entire AR system of *E. coli* and the complexity of its regulation have been extensively studied and well described in recent reviews^2^,^3^,^4^,^5^. Briefly, of the four clearly defined AR systems, AR1 is a RpoS-dependent oxidative (glucose-repressed) AR system that requires F_1_F_0_-ATPase, while its detailed molecular mechanism remains elusive^6^,^7^,^8^. The other two AR systems require amino acid decarboxylation^9^. The AR2 is a glutamate-dependent AR (GDAR) system that converts glutamate to γ-aminobutyric acid with decarboxylases (GadA and GadB)^10^,^11^,^12^ and imports glutamate in exchange for the γ-aminobutyric acid by selecting among protonated substrates using a charge-based mechanism of anti-porter (GadC)^13^. Similarly, the AR3 and AR4 are composed of arginine decarboxylase (AdiA) with the arginine/agmatine anti-porter (AdiC)^14^ and lysine decarboxylase (CadA) with lysine/cadaverine anti-porter (CadB)^15^, respectively. Consequently, this cycle pumps out cytoplasmic protons to the extracellular environment and reverses electrical membrane potential to slow proton movement into the cell, resulting in an increase of the intracellular pH to survive acidic stress. Of them, the AR2 is known to be the most effective system to relieve the acid stress^12^.

As mentioned above, the three genes (*gadA*, *gadB* and *gadC*) as well as several other genes in acid fitness islands (AFI) are mainly involved in the AR2 (GDAR) system; however, it is also under multiple levels of control. The GadE, GadW and GadX transcription factors (TFs) are at the first level of this regulatory cascade for direct regulation of these genes^16^,^17^,^18^. In addition, many other regulators such as PhoP^19^, YdeO^20^, EvgA^20^, TrmE^21^, RpoS^22^, RcsB^23^,^24^, Crp^25^, H-NS^26^,^27^ and TorR^28^ have been reported to affect induction of this AR system by forming a complex hierarchical regulatory cascade in response to different environmental signals. Although the general role of GadEWX in the regulation of the GDAR system and other genes in AFI has been extensively investigated with *in vitro* DNA-binding experiments, mutation analysis and comparative transcriptomics^16^,^17^,^18^,^27^,^29^,^30^,^31^,^32^,^33^,^34^,^35^,^36^,^37^, little is known about genome-scale *in vivo* GadEWX-binding events and the broader regulatory networks they comprise. A complete reconstruction of the GadEWX transcriptional regulatory networks under acidic stress would reveal the detailed mechanism of GadEWX regulation and its role in coordinating all cellular functions in response to acid stress.

In this study, we apply a systems biology approach to decode the GadEWX regulatory networks under acidic stress. We integrate genome-scale data from chromatin immunoprecipitation (ChIP) with lambda exonuclease digestion followed by high-throughput sequencing (ChIP-exo) for GadE, GadW, GadX and RpoS with those from strand-specific massively parallel cDNA sequencing (RNA-seq). To fully reconstruct the GadEWX regulons and RpoS involvement, we first examine the GadEWX- and RpoS-binding sites on the *E. coli* K-12 MG1655 genome. Then, we measure transcription levels of genes in the wild-type strain and knockout mutants of each TF on a genome scale to identify causal transcriptional regulatory relationships. From these data, we identify that the GadEWX regulatory networks maintain intracellular proton concentration by regulating proton-efflux/influx system and proton-generating/consuming metabolic enzymes and interconnecting the tree of them with pairs of negative-feedback loops. Reconstruction of the GadEWX regulatory networks provides a comprehensive view of the coordinative genome-wide regulatory roles of these TFs under acidic stress.

## Results

### Genome-wide binding profiles of GadEWX

Previously, several binding sites of GadEWX have been characterized in *E. coli* by *in vitro* DNA-binding experiments and mutation analysis^16^,^17^,^18^,^27^,^29^,^30^,^31^,^32^,^33^,^34^,^35^,^36^,^37^. However, direct measurement *in vivo* of GadEWX binding has not been achieved. Therefore, we employed a recently developed high-resolution ChIP-exo method to determine the *in vivo* genome-wide binding profiles of these three TFs with near 1-bp resolution in *E. coli* under acidic stress (pH 5.5).

**Figure 1:**
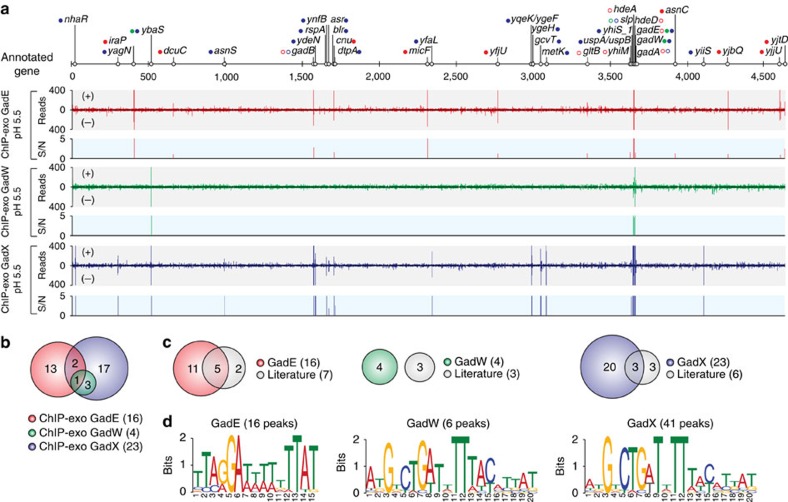
Genome-wide landscape of GadEWX-binding sites. (**a**) An overview of GadEWX-binding profiles across the *E. coli* genome (KiloBase) at mid-exponential growth phase (OD_600_=0.3) under acidic stress (pH 5.5). Open and closed dots indicate previously known and newly found GadEWX-binding sites, respectively. S/N denotes signal-to-noise ratio. (+) and (−) indicate reads mapped on forward and reverse strands, respectively. Binding peaks that overlap with Mock-IP signal were eliminated. Red, GadE; Green, GadW; Blue, GadX. (**b**) Overlaps between GadEWX-binding sites under the low-pH condition. (**c**) Comparison of the GadEWX binding sites obtained from this study (ChIP-exo) with the known binding sites from the literature. (**d**) Sequence logo representations of the GadEWX-DNA-binding motifs.

Using a peak finding algorithm (MACE program), 16, 6 and 41 reproducible binding peaks were identified for GadE, GadW and GadX, respectively, under acidic stress (Fig. 1a and Supplementary Tables 1–3). The number of binding sites of GadEWX was much lower than that of global TFs, suggesting that GadEWX may act more like local TFs to relieve acidic stress. In total, 16, 4 and 23 binding sites with multiple peaks were identified for GadE, GadW and GadX, respectively (Fig. 1b). We also found that the *in vivo* GadX binding sites include all of GadW binding sites as suggested in the previous comparative transcriptome study^17^. These numbers of binding sites and their overlap suggest that GadE and GadX play broader roles than GadW and that they have distinct regulatory networks from each other^32^. Before this study, 16 binding sites had been identified *in vitro* for GadEWX with strong experimental evidence, 50% (8 out of 16) of which were also detected in this study (Fig. 1c and Supplementary Table 4). It is unclear why these eight binding sites are missing from the data set obtained here. One possibility is that they are detectible only with *in vitro* methods and may not occur *in vivo* because of the interference by other regulators. Collectively, the identification of a total of 35 new binding sites was made possible by the new ChIP-exo method deployed here, significantly expanding the current knowledge of the scope of the GadEWX regulatory network.

We next assessed the widths and the genomic locations of the GadEWX-binding sites using currently available genome annotation. The widths of binding sites of each TF were 29±3.6, 30±7.6 and 28.7±8.0, respectively (Supplementary Fig. 1). The majority of GadEWX-binding sites (88%, or 38 out of 43) were observed within regulatory regions (that is, upstream of promoters, promoters and 5'-proximal to coding regions). The remaining 12% were found in intragenic regions or between two coding regions of convergent genes (Supplementary Tables 1–3). These results show a strong preference of GadEWX-binding site location being within noncoding intergenic regions with relatively fixed binding widths. To identify DNA sequence motifs of these newly identified GadEWX-binding sites, we used the sequence of each binding peak in the motif search procedure. The identified sequence motif of GadE from 16 binding peaks (TTARGAWWWWAAATA) was mostly consistent with the previously characterized asymmetric GadE-binding site (TTAGGAttTTgTTATTTAAa) (Fig. 1d)^16^. The motif search of GadW and GadX from 6 and 41 binding peaks, respectively, also yielded a sequence (AKGKCTGWTWTTWWYMYVAK), which resembled the previously reported binding motif (AtGtcTGATtTttatattat)^17^,^31^. This same motif is due to the binding regions of GadW being overlapped with those of GadX as revealed in the previous study^32^.

### Identification of GadEWX regulons and causal relationships

Currently, a total of 16 genes in 10 transcription units (TUs) (GadE: 12 genes in 7 TUs; GadW: 6 genes in 3 TUs; and GadX: 10 genes in 6 TUs) have been characterized with strong evidence as members of GadEWX regulons in *E. coli*^15^,^16^,^17^,^26^,^28^,^29^,^30^,^31^,^32^,^33^,^34^,^35^,^36^. Based on our ChIP-exo data sets, we can significantly expand the size of the GadEWX regulons to comprise 45 target genes in 31 TUs (GadE: 20 genes in 15 TUs; GadW: 6 genes in 4 TUs; GadX: 29 genes in 19 TUs, several genes are co-regulated) (Supplementary Tables 1–3). Functional analysis with clusters of orthologous groups (COG) categories was performed to look for any functional enrichment of GadEWX regulon genes (Supplementary Fig. 2). The COG category for amino acid metabolism and transport (E) was found to be statistically overrepresented (hypergeometric test *P*-value=4 × 10^−6^) and it includes *gadA*, *gadBC*, *gltBD* and *ybaST*, whose reactions are closely related with glutamate metabolism. Besides, a diverse range of COG functional categories indicate that GadEWX may play complicated roles beyond regulating genes in AFI to coordinate associated cellular processes in *E. coli* K-12 MG1655 as GadE has multiple roles in other pathogenic *E. coli* strains^38^,^39^,^40^. We in addition analysed what other TFs are known to regulate these 45 genes based on the strong evidence in RegulonDB^41^ and found that 21 of them are regulated by 22 other TFs (Supplementary Table 5). Interestingly, the core genes in the GDAR system (*gadA* and *gadBC*) were regulated by three additional TFs (H-NS, FliZ and AdiY)^42^,^43^. This result represents the importance of complicated regulation of these genes according to the changes of the environments. The remaining 24 genes were not directly regulated by other TFs, meaning that GadEWX exclusively regulates these genes in response to acid stress.

To determine the causal relationships between the binding of GadEWX and changes in RNA transcript levels of genes in the GadEWX regulons, we compared transcript levels between the wild-type strain and that of each deletion mutant (Δ*gadE*, Δ*gadW* and Δ*gadX*) grown under acidic stress conditions. Overall, a total of 351 genes were differentially expressed in at least one mutant. Genes with expression changes with log2 fold change≥0.5 and false discovery rate <0.01 were defined as differentially expressed. Only 25 out of 351 were differentially expressed in all mutants (Supplementary Fig. 3 and Supplementary Data 1–3). This unexpectedly small overlap in the transcriptional response suggests that each TF has a specific transcriptional response and may have a distinct role in regulating the AR system.

**Figure 2:**
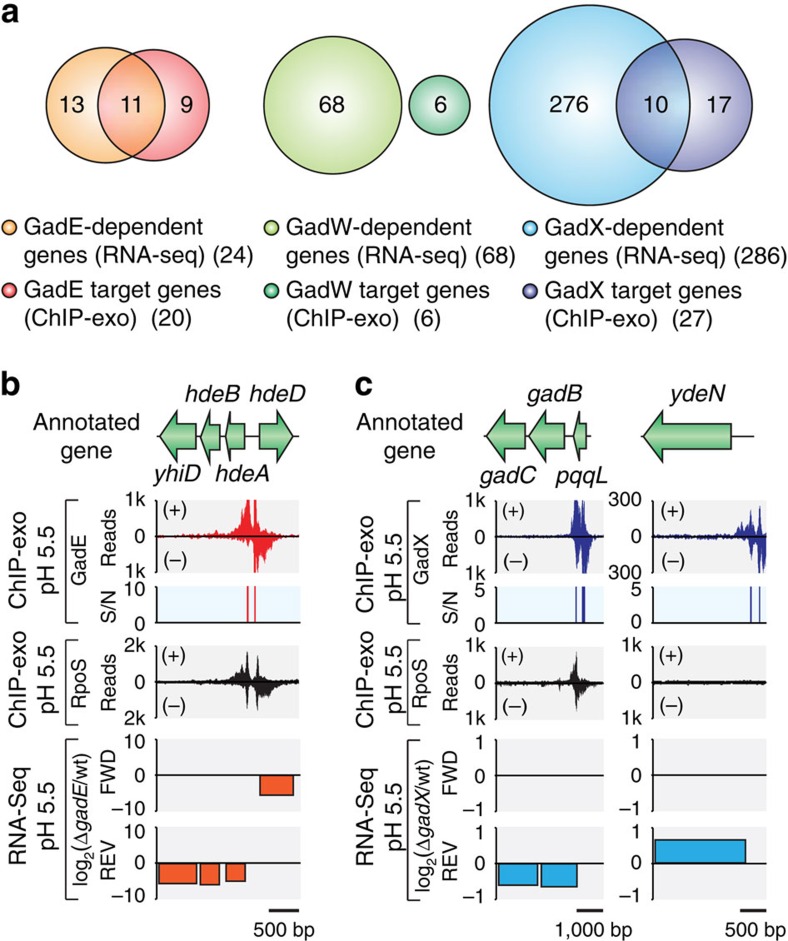
Genome-wide identification of GadEWX regulons and RpoS involvement. (**a**) Comparison of ChIP-exo results and gene expression profiles to define direct GadEWX regulons. Examples of (**b**) GadE regulon (*hdeAB-yhiD* and *hdeD* with divergent promoters) and (**c**) GadX regulon (*gadBC* and *ydeN*). S/N denotes signal-to-noise ratio. (+) and (−) in ChIP-exo data indicate reads mapped on forward and reverse strands, respectively.

RpoS, one of the alternative sigma factors in *E. coli*, is known to be crucial for expression of GadEWX and GDAR as well as performing general stress management in association with EvgS/EvgA and PhoQ/PhoP^44^,^45^,^46^. To investigate the involvement of RpoS in regulating GadEWX regulons, we additionally performed ChIP-exo to determine the *in vivo* binding profile of RpoS under acid stress. Combining our GadEWX- and RpoS-binding maps with a GadEWX-dependent transcriptome, we could determine the causal relationships between the binding of GadEWX and the changes in transcript levels of genes in GadEWX regulons under acidic stress as well as RpoS involvement for expression (Fig. 2). Among 45 target genes identified from ChIP-exo analysis of GadEWX, we determined that 19 genes in 13 TUs (GadE, 11 genes in 7 TUs; GadX, 10 genes in 7 TUs; *gadBC* are overlapped) (43%) were directly regulated by GadEWX under acidic stress (Fig. 2a, Supplementary Fig. 4 and Supplementary Tables 1–3). In addition, the expression of 62% of GadEWX regulon members (28 out of 45) was involved in the association of RpoS (Supplementary Fig. 4 and Supplementary Tables 1–3). Among the binding events of RpoS on GadEWX regulon members, 64% of them (18 out of 28) were novel (Supplementary Fig. 4 and Supplementary Tables 1–3). This result confirms the importance of RpoS recruitment for acid-stress response and expands the current knowledge of RpoS involvement in *E. coli*.

For example, the divergent promoters upstream of *hdeA* and *hdeD* TUs were extensively occupied by GadE and RpoS, and this GadE binding significantly increased transcript level of downstream genes under the acidic condition (Fig. 2b). Likewise, GadE acted as an activator on other promoters (Supplementary Table 1). The calculation of relative distance from GadE bindings to transcription start site^41^,^47^ showed that GadE binding sites are localized to either further upstream or downstream of the promoter region (−35 and −10 boxes) (Supplementary Table 1). This observation suggests that GadE may prevent binding of other repressors or directly activate transcription through another mechanism, such as recruiting RNAP to activate transcription^32^. However, unlike GadE, GadX acted as a dual regulator either to activate or repress the transcription of target genes. For instance, the association of GadX and RpoS on *gadB* promoter increased transcript level under acidic stress (Fig. 2c). However, the binding of GadX on *ydeN* promoter decreased transcript level and RpoS association was not observed (Fig. 2d). Although GadW bound to the promoter regions (Supplementary Table 2), we were not able to observe significant changes in transcript levels upon *gadW* knockout as shown in the previous transcriptome study^17^. Also, the remaining 59% of the genes (including GadW target genes) lacked significant changes in transcript levels despite the binding of corresponding TFs. These results indicate that the changes in their transcript levels may require additional regulatory signals such as association or dissociation of other TFs.

### Genome-wide roles of GadEWX regulons

**Figure 3:**
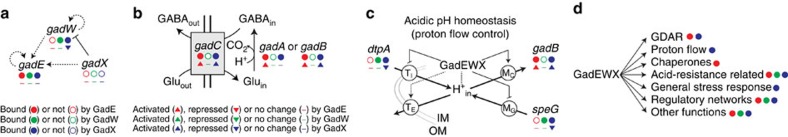
Genome-wide roles of the GadEWX regulons in *E. coli*. Regulation of (**a**) GadEWX regulatory circuits and (**b**) core genes in the glutamate-dependent acid resistance (GDAR) system. Closed and open circles represent whether target genes are bound by TF or not. Triangle, inverted triangle, and dash indicate whether the expression levels of target genes are activated, repressed, or not changed upon TF knockout. Red, GadE; Green, GadW; Blue, GadX. (**c**) Schematic diagram for the reconstructed GadEWX regulatory motif for acidic pH homeostasis. The GadEWX repressed proton-influx system (T_I_) and proton-generating metabolic enzymes (M_G_) while activated proton-consuming metabolic enzymes (M_C_). We were not able to observe regulation of proton-efflux system (T_E_) by GadEWX. IM, inner membrane; OM, outer membrane. (**d**) GadEWX regulatory networks regulate diverse cellular functions. Arrows with dotted lines indicate that the regulation is not observed in this study.

To determine how GadEWX coherently regulate gene expression in response to acidic stress, we reconstructed the genome-wide GadEWX regulatory network in *E. coli*. First, we looked into the regulatory circuit that GadEWX comprises. Although we detected bindings of each TF on either itself or other TFs as known previously^2^, we were not able to observe changes of transcript levels by association of these TFs except for the repression of *gadW* by GadX (Fig. 3a). Since we compared transcript levels between wild-type and each of deletion mutant (Δ*gadE*, Δ*gadW* or Δ*gadX*) to determine causal relationships, we are not able to examine the autoregulation of TFs. A previous study showed that the transcription of *gadE* is activated by GadW and GadX when cells were grown to stationary phase at pH 5.5 in LB glucose media^18^. However, a different growth phase or growth media could have contributed to different transcriptional regulation on *gadE*. (Fig. 3a and Supplementary Tables 2 and 3). We believe that multiple reiterative-control circuits consisting of other regulators still activate GadE, which is crucial in the GDAR system for AR, even if GadW or GadX is deleted^48^.

A recent kinetic study of promoters and regulators in the AR2 system claimed that GadX (*gadXW* double mutation) did not affect the kinetics of the promoters of core genes (*gadA* and *gadBC*) in the GDAR system^48^. However, another comparative transcriptome analysis suggested that these promoters are significantly activated by GadX^17^. Interestingly, our study observed that both GadE and GadX activate transcription of these core genes, but with a different activation ratio (over 50-fold activation by GadE but less than 2-fold by GadX; Supplementary Data 1 and 3). These results demonstrate that GadE is a much stronger activator than GadX and consequently GadX cannot fully compensate for the loss of activation of the GDAR system caused by *gadE* deletion. Similarly, physiological studies showing that the *gadE* mutant was more sensitive to acid challenge than the *gadX* mutant also supported this observation^16^,^17^,^49^.

The comprehensive genome-wide reconstruction of the GadEWX regulatory network in *E. coli* allowed us to extend the scope of its roles under acidic stress. After functional classification of 43 genes out of 45 GadEWX regulons excluding *gadEW*, we observed that the functions of 23% (10) of those genes were mainly involved in proton consumption, generation and transport. Under acidic stress, only GadX-repressed genes involved in proton generation and influx such as *speG* (spermidine acetyltransferase) and *dtpA* (proton-dependent oligopeptide transporter) (Fig. 3c). On the other hand, both GadE and GadX activated several genes, such as *gadA* and *gadB* that encode enzyme consuming protons. We could not observe any proton-efflux systems regulated by GadEWX in *E. coli* K-12 MG1655. From this analysis, we believe that GadEWX connect proton-efflux/influx and generating/consuming enzymes with negative-feedback loop pairs to maintain intracellular proton concentration for pH homeostasis (Fig. 3c).

In addition to the control of proton flow, GadE directly regulated the expression of chaperone-encoding genes (*hdeA* and *hdeB*)^5^. Both GadE and GadX participated in controlling the expression of other acid-resistance related genes (*asr*, *slp*, *yhiM*, *yhiD* and *hdeD*) known to enhance the survival of cells under acid stress^17^,^49^,^50^ (Fig. 3d and Supplementary Fig. 4). Based on genome-wide binding properties, GadX was likely to participate in the regulation of general stress response (*uspB*, *uspA* and *uspD*). Interestingly, GadEWX were coordinated with other regulatory networks (*iraP*, *cnu*, *micF*, *nhaR*, *asnC* and *dctR*) indicating that GadEWX may play more complex genome-wide roles in association with other TFs. Several genes with predicted or unknown functions (*yjbQ*, *yjbR*, *yjjU*, *yjtD*, *yiiS*, *ynfB*, *yfaL*, *yqeK*, *ygeF* and *ygeH*) were also regulated by GadE and GadX (Fig. 3d). Recent studies revealed that expression of genes of the locus of enterocyte effacement (LEE) for pathogenesis of *E. coli* O157:H7 was indirectly regulated by GadE and GadX^39^,^40^. In *E. coli* K-12 MG1655, we found GadX binding peaks (*yqeK*, *ygeF* and *ygeH*) within a remnant of a pathogenicity island (Supplementary Table 3). Since it was found that GadE and GadX indirectly regulate LEE in *E. coli* O157:H7 (ref. 39), investigation of genome-wide *in vivo* bindings of these regulators in pathogenic strains might be able to reveal regulatory cascades in LEE that cause pathogenicity. To further examine the physiological roles of GadEWX regulon members with predicted or unknown functions under acid stress, we chose five genes (*yjbQ*, *yjbR*, *yjjU*, *yjtD* and *yiiS*) that either were activated or showed no causal relationship upon single TF knockout. Acid-stress resistance assays of knockout strains of these genes revealed that they were not directly related to GDAR, but three of them (*yjjU*, *yjtD* and *yiiS*) were implicated in general AR (Supplementary Fig. 5). This result indicates that GadEWX contribute to generate more global acid-resistance responses in *E. coli* beyond the regulation of the GDAR system.

### Evolutionary aspects of GadEWX regulons

**Figure 4:**
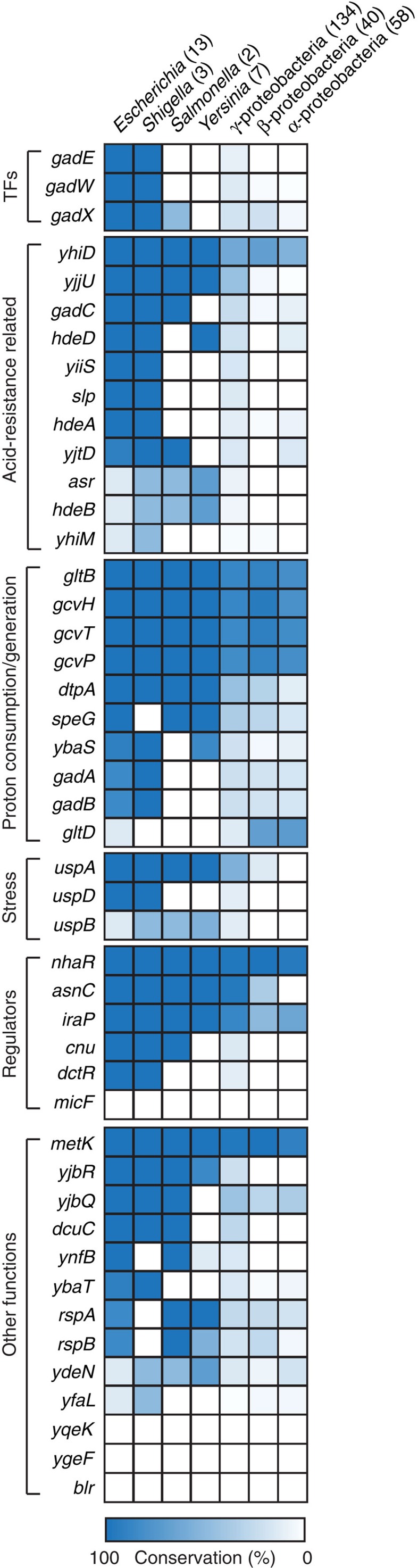
Evolution of GadEWX regulons. Conservation rates of GadEWX regulons across γ-, β-, α-proteobacteria are presented based on the ortholog calculation. The genes are subdivided by their functional categories.

A recent survey on conservation analysis of the *gadBC* system in mostly enteric bacteria suggested that the presence and expression of this operon in pathogenic bacteria has been strongly connected to the requirement to survive under extremely acidic conditions but not necessarily in other non-pathogenic bacteria^12^. We further expanded our investigation of how *E. coli* GadEWX and their entire regulons have been evolved in the proteobacteria phylum including 134 γ-proteobacteria, 40 β-proteobacteria and 58 α-proteobacteria. We first compared the conservation of the genes encoding the regulators (*gadEWX*). They were almost conserved in *Escherichia* and *Shigella*, but not in *Salmonella*, *Yersinia* and even further distant enterobacteria (Fig. 4). As stated in the previous studies^12^,^51^, the presence of the GDAR system (*gadA*, *gadB* and *gadC*) in these bacteria would allow them to survive in an extreme acidic environment (pH 2) in the presence of glutamate. Interestingly, *gadX* as well as *gadAB* are conserved in some of β- and α-proteobacteria whereas *gadE* and *gadW* are not. Probably, GadX alone would be able to regulate the GDAR system in part when GadE is absent in these classes. Genes related to the proton consumption/generation (*gltBD*, *gcvHTP*, *ybaS* and so on) exhibited higher conservation ratio across proteobacteria even in the species that do not have the GDAR system (Fig. 4). These genes may be regulated by other TFs and participate in controlling intracellular proton flow.

By maintaining pH homeostasis under alkaline condition, the Na^+^/H^+^ anti-porter plays a crucial role in adaptation under alkaline challenge^4^. It should be noted that *nhaR* encoding Na^+^/H^+^ anti-porter activator was strongly bound by GadX and RpoS under acidic stress (Supplementary Table 3). Since the deletion of GadX did not significantly affect the transcription level of *nhaR*, additional signal and/or TF associations may be required in regulation of this activator under acidic condition so that it can be activated only under alkaline condition. Unlike the GDAR system, it seems like the involvement of cation–proton anti-porter is a commonly used strategy for alkaline challenge across proteobacteria given the high conservation rate of *nhaR* (Fig. 4).

## Discussion

We comprehensively reconstructed the GadEWX regulons at the genome scale in *E. coli* by combining genome-wide GadEWX-binding maps and GadEWX-dependent transcriptome daunder acidic stress. We identified (i) a total of 45 genes in 31 TUs that belong to GadEWX regulons and associations of RpoS for 28 of those genes; (ii) 19 genes in 13 TUs showing causal relationships upon single TF knockout; (iii) the negative-feedback loop pairs of proton transporters and utilization enzymes by GadEWX for pH homeostasis; and (iv) the additional roles of GadEWX regulatory networks beyond the GDAR system.

Using active proton transports^4^ is a major strategy for bacterial pH homeostasis. They include the proton-pumping respiratory chain complexes such as cytochrome *bo* terminal oxidase (cbo) and NADH:ubiquinone oxidoreductase (*ndh*). Under conditions of acid challenge, *E. coli* increases expression of those complexes that pump protons out of the cell. Our recently developed genome-scale model of metabolism and gene expression (ME-Model) in *E. coli*^52^ predicted that these proton-pumping complexes are more efficient for relieving the acid stress than other amino acid decarboxylase-anti-porter pairs (AR2 and AR3) (Supplementary Fig. 6). Nevertheless, AR2 and AR3 are certainly required by cells for survival when passing through extreme acidic environments such as the stomach as they secrete cytoplasmic protons to the extracellular environment^53^,^54^.

Recently, two studies showed interesting observations on AR in *E. coli*. One study revealed that polyamines such as spermidine, putrescine and cadaverine induce multiple components in GDAR and they are important for protection against acidic stress^55^. Surprisingly, we also found that GadX represses RpoS-involved transcription of *speG* (spermidine acetyltransferase), which consequently controls the polyamine concentration by degradation (Fig. 3c and Supplementary Fig. 4). This reaction also generates protons during acetylation of spermidine which agrees with our hypothesis that proton-generating enzymes will be repressed for the control of proton flow. By doing so, cells can regulate the level of intracellular proton and polyamine concentration, so that they can survive under acidic stress. The other study reported that enzymatic release of ammonia by *ybaS* encoding glutaminase neutralizes protons, resulting in elevated intracellular pH under acidic environment^53^. We identified that GadW, GadX and RpoS bound to the promoter region of *ybaS* while we could not observe significant changes in transcript level upon single TF knockout (Supplementary Fig. 4). However, a previous microarray-based study showed that the double knockout strain (Δ*gadXW*) showed significant changes in transcript level of *ybaS* compared to wild type^17^, indicating that transcription of *ybaS* can be activated in the presence of either GadW or GadX. In addition, the promoter region of *gcvHTP* encoding glycine cleavage system, which is known to release ammonia similar to *ybaS*, was also occupied by GadX (Supplementary Table 3). Given the properties of the *ybaS* system, it is plausible that this system may also contribute to AR by releasing ammonia. In summary, we have described the complex roles of the GadEWX regulatory network in *E. coli* under acidic stress by a systems approach that integrates various types of cutting-edge genome-scale experimental data. Future efforts on revealing the precise molecular role of small RNAs in GadEWX transcriptional regulatory networks by recently developed techniques such as ribosome profiling^56^ would expand acid-response regulatory networks including the post-transcription level. Understanding AR in microbes has important implications for prevention and clinical treatment as well as health-care applications such as probiotics^57^ and even for engineering microbes to produce organic acids^58^. Further, similar to how the genome-scale model of *E. coli* metabolism with protein structures (GEM-PRO)^59^ successfully predicted thermostability of proteomes, the inclusion of structural information under acidic stress with these comprehensive operon structures into the current model^52^ would expand the predictive capability of the model on future complex phenotypes.

## Methods

### Bacterial strains and growth conditions

All strains used are *E. coli* K-12 MG1655 and its derivatives. The *E. coli* strains harbouring GadE-8myc, GadW-8myc and GadX-8myc were generated by a λ Red-mediated site-specific recombination system targeting C-terminal region of each gene^60^. Deletion mutants (Δ*gadX*, Δ*gadW*, Δ*gadX* and other *y-genes*) were also constructed by a λ Red-mediated site-specific recombination system^61^. Glycerol stocks of *E. coli* strains were inoculated into fresh 70 ml of M9 minimal medium (47.8 mM Na_2_HPO_4_, 22 mM KH_2_PO_4_, 8.6 mM NaCl, 18.7 mM NH_4_Cl, 2 mM MgSO_4_ and 0.1 mM CaCl_2_) supplemented with 0.2% (w/v) glucose in 500 ml flask and cultured overnight at 37 ^o^C with 250 r.p.m. For acidic stress, the overnight cultures were inoculated into the fresh 70 ml of M9 minimal medium at pH 5.5 (adjusted with HCl) in 500 ml flask and continued to culture at 37^o^C with 250 r.p.m. to OD_600_=0.3±0.02. At the sampling point, the pH was measured to be 5.28±0.03.

### ChIP-exo

To identify GadEWX- and RpoS-binding maps *in vivo*, we isolated the DNA bound to each protein from formaldehyde cross-linked *E. coli* cells by ChIP with the specific antibodies that specifically recognizes myc tag (9E10, Santa Cruz Biotechnology) and RpoS (1RS1, Neoclone) with 1:50 dilutions, and Dynabeads Pan Mouse IgG magnetic beads (Invitrogen) were also used to capture specific antibody followed by stringent washings^62^. With ChIP materials (chromatin-beads), we performed on-bead enzymatic reactions of the original ChIP-exo method^63^ with following modifications as shown in our previous study^64^. Briefly, the sheared DNA of chromatin-beads was end-repaired, dA-tailed and ligated with the first adaptor (5'-phosphorylated). After that, nick sites of each fragment were repaired before Lambda exonuclease and RecJ_f_ exonuclease treatment. The protein-DNA crosslink was reversed by overnight incubation at 65 ^°^C with RNAs and proteins removal. DNA samples were used to perform primer extension and second adaptor ligation. The size-selected DNA sample was amplified and quantified. Prepared DNA library was sequenced using MiSeq (Illumina) in accordance with the manufacturer's instructions. ChIP-exo experiments were performed in biological duplicate. Sequence reads generated from ChIP-exo were mapped onto the reference genome (NC_000913.2) using bowtie^65^ with default options to generate SAM output files. MACE program ( https://code.google.com/p/chip-exo/)^66^-based in-house script was used to define peak candidates from biological duplicates with sequence depth normalization. To reduce false-positive peaks, peaks with signal-to-noise ratio less than 1.0 and same signal with Mock-IP were removed as in our previous study^64^.

### RNA-seq expression profiling

Total RNAs including small RNAs were isolated using the cells treated with RNAprotect Bacteria Reagent (Qiagen) at OD_600_=0.3±0.02 followed by purification steps using Qiagen RNeasy Plus Mini Kit (Qiagen) in accordance to the manufacturer's instruction to collect both of them. Strand-specific RNA-seq library was prepared using the dUTP method^67^ with the following modifications as shown in our previous study^64^. Briefly, the rRNA-removed RNA sample was fragmented, and then first and second strands were synthesized. The samples were sequenced using MiSeq (Illumina) in accordance with the manufacturer's instructions. RNA-seq experiments were performed in biological duplicate. Sequence reads generated from RNA-seq were mapped onto the reference genome (NC_000913.2) using bowtie^65^ with the maximum insert size of 1000, bp, and 2 maximum mismatches after trimming 3 bp at 3' ends. These files were used for Cufflinks ( http://cufflinks.cbcb.umd.edu/)^68^ and Cuffdiff to calculate fragments per kilobase of exon per million fragments and differential expression, respectively. Cufflinks and Cuffdiff were run with default options with library type of dUTP RNA-seq. The RNA-seq data generated by our study were reproducible when comparing fragments per kilobase of exon per million fragments values of all genes and sRNAs between replicates (Supplementary Fig. 7). From cuffdiff output, genes with differential expression with log2 fold change ≥0.5 and a false discovery rate value<0.01 were considered as differentially expressed genes.

### Motif search and analysis

The GadEWX-binding motif analyses were completed using the MEME tool from the MEME software suite with default settings^69^. We extended the sequence of each binding site by 10–30 bp at each end to allow for adjacent sequences to be included in the analysis.

### COG functional enrichment

The GadEWX regulons were categorized according to their annotated COG category. Functional enrichment of COG categories was determined by performing one-tailed Fisher's exact test (Hypergeometric test), and *P-value*<0.05 was considered significant.

### Acid resistance assays

AR assays followed the procedures of previous study with modification of usage of growth media^49^. Cells grown for overnight in M9 minimal media (pH 7.0) were diluted into fresh M9 media at pH 5.5 and cultured to OD_600_=0.3. These adapted cell cultures were inoculated into M9 minimal media at pH 2.5 without or with 1.5 mM sodium glutamate. The initial cell density inoculated for acid challenge was between 1 × 10^6^ and 3 × 10^6^ colony-forming unit (CFU) per ml. The pH 2.5 cultures were then incubated at 37 °C without shaking, and samples were collected after 2 h. Aliquots were serially diluted, and triplicates were plated onto LB agar plates. Colonies were counted after 24 h. Percent survival was calculated as follows^49^: ((CFU per ml at time 2 h)/(CFU per ml at time zero)) × 100. The results presented are averages of triplicate experiments and include the standard deviations.

### Conservation analysis of GadEWX regulons

Gene annotation of strains and species were obtained from the SEED server ( http://theseed.org) and ortholog calculation to *E. coli* K-12 MG1655 was also performed on RAST (Rapid Annotation using Subsystem Technology) server^70^. Conservation level of *gadEWX* and genes in GadEWX regulons were calculated from orthologs retained from RAST output.

## Additional information

**Accession codes:** All raw data for ChIP-exo and RNA-seq has been deposited into the Gene Expression Omnibus at the National Center for Biotechnology Information under the accession code GSE66482.

**How to cite this article:** Seo, S.W. *et al.* Decoding genome-wide GadEWX-transcriptional regulatory networks reveals multifaceted cellular responses to acid stress in *Escherichia coli*. *Nat. Commun.* 6:7970 doi: 10.1038/ncomms8970 (2015).

## Supplementary Material

Supplementary Information Supplementary Figures 1-7 and Supplementary Tables 1-5

Supplementary Data 1 Differentially expressed genes (DEGs) due to *gadE* deletion under acidic stress

Supplementary Data 2 Differentially expressed genes (DEGs) due to *gadW* deletion under acidic stress

Supplementary Data 3 Differentially expressed genes (DEGs) due to *gadX* deletion under acidic stress

## References

[b1] Talley K. & Alexov E. On the pH-optimum of activity and stability of proteins. Proteins 78, 2699–2706 (2010). 20589630 10.1002/prot.22786 PMC2911520

[b2] Foster J. W. *Escherichia coli* acid resistance: tales of an amateur acidophile. Nat. Rev. Microbiol. 2, 898–907 (2004). 15494746 10.1038/nrmicro1021

[b3] Lund P., Tramonti A. & De Biase D. Coping with low pH: molecular strategies in neutralophilic bacteria. FEMS Microbiol. Rev. 38, 1091–1125 (2014). 24898062 10.1111/1574-6976.12076

[b4] Krulwich T. A., Sachs G. & Padan E. Molecular aspects of bacterial pH sensing and homeostasis. Nat. Rev. Microbiol. 9, 330–343 (2011). 21464825 10.1038/nrmicro2549 PMC3247762

[b5] Hong W., Wu Y. E., Fu X. & Chang Z. Chaperone-dependent mechanisms for acid resistance in enteric bacteria. Trends. Microbiol. 20, 328–335 (2012). 22459131 10.1016/j.tim.2012.03.001

[b6] Castanie-Cornet M. P. *et al.* Control of acid resistance in *Escherichia coli*. J. Bacteriol. 181, 3525–3535 (1999). 10348866 10.1128/jb.181.11.3525-3535.1999 PMC93821

[b7] Lin J. *et al.* Comparative analysis of extreme acid survival in *Salmonella typhimurium*, *Shigella flexneri*, and *Escherichia coli*. J. Bacteriol. 177, 4097–4104 (1995). 7608084 10.1128/jb.177.14.4097-4104.1995 PMC177142

[b8] Sun Y., Fukamachi T., Saito H. & Kobayashi H. Respiration and the F(1)Fo-ATPase enhance survival under acidic conditions in *Escherichia coli*. PLoS ONE 7, e52577 (2012). 23300708 10.1371/journal.pone.0052577 PMC3534200

[b9] Diez-Gonzalez F. & Karaibrahimoglu Y. Comparison of the glutamate-, arginine- and lysine-dependent acid resistance systems in *Escherichia coli* O157:H7. J. Appl. Microbiol. 96, 1237–1244 (2004). 15139915 10.1111/j.1365-2672.2004.02251.x

[b10] Hersh B. M. *et al.* A glutamate-dependent acid resistance gene in *Escherichia coli*. J. Bacteriol. 178, 3978–3981 (1996). 8682809 10.1128/jb.178.13.3978-3981.1996 PMC232665

[b11] Lin J. *et al.* Mechanisms of acid resistance in enterohemorrhagic *Escherichia coli*. Appl. Environ. Microbiol. 62, 3094–3100 (1996). 8795195 10.1128/aem.62.9.3094-3100.1996 PMC168100

[b12] De Biase D. & Pennacchietti E. Glutamate decarboxylase-dependent acid resistance in orally acquired bacteria: function, distribution and biomedical implications of the gadBC operon. Mol. Microbiol. 86, 770–786 (2012). 22995042 10.1111/mmi.12020

[b13] Tsai M. F., McCarthy P. & Miller C. Substrate selectivity in glutamate-dependent acid resistance in enteric bacteria. Proc. Natl Acad. Sci. USA 110, 5898–5902 (2013). 23530225 10.1073/pnas.1301444110 PMC3625338

[b14] Fang Y., Kolmakova-Partensky L. & Miller C. A bacterial arginine-agmatine exchange transporter involved in extreme acid resistance. J. Biol. Chem. 282, 176–182 (2007). 17099215 10.1074/jbc.M610075200

[b15] Meng S. Y. & Bennett G. N. Nucleotide sequence of the *Escherichia coli* cad operon: a system for neutralization of low extracellular pH. J. Bacteriol. 174, 2659–2669 (1992). 1556085 10.1128/jb.174.8.2659-2669.1992 PMC205906

[b16] Ma Z. *et al.* GadE (YhiE) activates glutamate decarboxylase-dependent acid resistance in *Escherichia coli* K-12. Mol. Microbiol. 49, 1309–1320 (2003). 12940989 10.1046/j.1365-2958.2003.03633.x

[b17] Tucker D. L. *et al.* Genes of the GadX-GadW regulon in *Escherichia coli*. J. Bacteriol. 185, 3190–3201 (2003). 12730179 10.1128/JB.185.10.3190-3201.2003 PMC154079

[b18] Sayed A. K., Odom C. & Foster J. W. The *Escherichia coli* AraC-family regulators GadX and GadW activate gadE, the central activator of glutamate-dependent acid resistance. Microbiology 153, 2584–2592 (2007). 17660422 10.1099/mic.0.2007/007005-0

[b19] Zwir I. *et al.* Dissecting the PhoP regulatory network of *Escherichia coli* and *Salmonella enterica*. Proc. Natl Acad. Sci. USA 102, 2862–2867 (2005). 15703297 10.1073/pnas.0408238102 PMC548500

[b20] Masuda N. & Church G. M. Regulatory network of acid resistance genes in *Escherichia coli*. Mol. Microbiol. 48, 699–712 (2003). 12694615 10.1046/j.1365-2958.2003.03477.x

[b21] Cabedo H. *et al.* The *Escherichia coli* trmE (mnmE) gene, involved in tRNA modification, codes for an evolutionarily conserved GTPase with unusual biochemical properties. EMBO J. 18, 7063–7076 (1999). 10601028 10.1093/emboj/18.24.7063 PMC1171769

[b22] De Biase D., Tramonti A., Bossa F. & Visca P. The response to stationary-phase stress conditions in *Escherichia coli*: role and regulation of the glutamic acid decarboxylase system. Mol. Microbiol. 32, 1198–1211 (1999). 10383761 10.1046/j.1365-2958.1999.01430.x

[b23] Johnson M. D. *et al.* RcsB is required for inducible acid resistance in *Escherichia coli* and acts at gadE-dependent and -independent promoters. J. Bacteriol. 193, 3653–3656 (2011). 21571995 10.1128/JB.05040-11 PMC3133336

[b24] Castanie-Cornet M. P. *et al.* The glutamate-dependent acid resistance system in *Escherichia coli*: essential and dual role of the His-Asp phosphorelay RcsCDB/AF. Microbiology. 153, 238–246 (2007). 17185552 10.1099/mic.0.29278-0

[b25] Ma Z., Richard H. & Foster J. W. pH-Dependent modulation of cyclic AMP levels and GadW-dependent repression of RpoS affect synthesis of the GadX regulator and *Escherichia coli* acid resistance. J. Bacteriol. 185, 6852–6859 (2003). 14617649 10.1128/JB.185.23.6852-6859.2003 PMC262709

[b26] De Biase D., Tramonti A., Bossa F. & Visca P. The response to stationary-phase stress conditions in *Escherichia coli*: role and regulation of the glutamic acid decarboxylase system. Mol. Microbiol. 32, 1198–1211 (1999). 10383761 10.1046/j.1365-2958.1999.01430.x

[b27] Tramonti A. *et al.* Functional characterization and regulation of gadX, a gene encoding an AraC/XylS-like transcriptional activator of the *Escherichia coli* glutamic acid decarboxylase system. J. Bacteriol. 184, 2603–2613 (2002). 11976288 10.1128/JB.184.10.2603-2613.2002 PMC135039

[b28] Bordi C., Theraulaz L., Mejean V. & Jourlin-Castelli C. Anticipating an alkaline stress through the Tor phosphorelay system in *Escherichia coli*. Mol. Microbiol. 48, 211–223 (2003). 12657056 10.1046/j.1365-2958.2003.03428.x

[b29] Ma Z. *et al.* Collaborative regulation of *Escherichia coli* glutamate-dependent acid resistance by two AraC-like regulators, GadX and GadW (YhiW). J. Bacteriol. 184, 7001–7012 (2002). 12446650 10.1128/JB.184.24.7001-7012.2002 PMC135476

[b30] Sayed A. K. & Foster J. W. A 750bp sensory integration region directs global control of the *Escherichia coli* GadE acid resistance regulator. Mol. Microbiol. 71, 1435–1450 (2009). 19220752 10.1111/j.1365-2958.2009.06614.x

[b31] Tramonti A., De Canio M. & De Biase D. GadX/GadW-dependent regulation of the *Escherichia coli* acid fitness island: transcriptional control at the gadY-gadW divergent promoters and identification of four novel 42bp GadX/GadW-specific binding sites. Mol. Microbiol. 70, 965–982 (2008). 18808381 10.1111/j.1365-2958.2008.06458.x

[b32] Tramonti A. *et al.* Mechanisms of transcription activation exerted by GadX and GadW at the gadA and gadBC gene promoters of the glutamate-based acid resistance system in *Escherichia coli*. J. Bacteriol. 188, 8118–8127 (2006). 16980449 10.1128/JB.01044-06 PMC1698215

[b33] Giangrossi M. *et al.* Antagonistic role of H-NS and GadX in the regulation of the glutamate decarboxylase-dependent acid resistance system in *Escherichia coli*. J. Biol. Chem. 280, 21498–21505 (2005). 15795232 10.1074/jbc.M413255200

[b34] Opdyke J. A., Kang J. G. & Storz G. GadY, a small-RNA regulator of acid response genes in *Escherichia coli*. J. Bacteriol. 186, 6698–6705 (2004). 15466020 10.1128/JB.186.20.6698-6705.2004 PMC522195

[b35] Hommais F. *et al.* GadE (YhiE): a novel activator involved in the response to acid environment in *Escherichia coli*. Microbiology. 150, 61–72 (2004). 14702398 10.1099/mic.0.26659-0

[b36] Tramonti A., De Canio M., Bossa F. & De Biase D. Stability and oligomerization of recombinant GadX, a transcriptional activator of the *Escherichia coli* glutamate decarboxylase system. Biochim. Biophys. Acta 1647, 376–380 (2003). 12686161 10.1016/s1570-9639(03)00098-0

[b37] Castanie-Cornet M. P. & Foster J. W. *Escherichia coli* acid resistance: cAMP receptor protein and a 20bp cis-acting sequence control pH and stationary phase expression of the gadA and gadBC glutamate decarboxylase genes. Microbiology 147, 709–715 (2001). 11238978 10.1099/00221287-147-3-709

[b38] Tree J. J. *et al.* Transcriptional regulators of the GAD acid stress island are carried by effector protein-encoding prophages and indirectly control type III secretion in enterohemorrhagic *Escherichia coli* O157:H7. Mol. Microbiol. 80, 1349–1365 (2011). 21492263 10.1111/j.1365-2958.2011.07650.x PMC7099609

[b39] Branchu P. *et al.* NsrR, GadE, and GadX interplay in repressing expression of the *Escherichia coli* O157:H7 LEE pathogenicity island in response to nitric oxide. PLoS. Pathog. 10, e1003874 (2014). 24415940 10.1371/journal.ppat.1003874 PMC3887101

[b40] Kailasan Vanaja S., Bergholz T. M. & Whittam T. S. Characterization of the *Escherichia coli* O157:H7 Sakai GadE regulon. J. Bacteriol. 191, 1868–1877 (2009). 19114477 10.1128/JB.01481-08 PMC2648353

[b41] Salgado H. *et al.* RegulonDB v8.0: omics data sets, evolutionary conservation, regulatory phrases, cross-validated gold standards and more. Nucleic Acids Res. 41, D203–D213 (2013). 23203884 10.1093/nar/gks1201 PMC3531196

[b42] Yoshida T., Yamashino T., Ueguchi C. & Mizuno T. Expression of the *Escherichia coli* dimorphic glutamic acid decarboxylases is regulated by the nucleoid protein H-NS. Biosci. Biotechnol. Biochem. 57, 1568–1569 (1993). 7764225 10.1271/bbb.57.1568

[b43] Krin E., Danchin A. & Soutourina O. Decrypting the H-NS-dependent regulatory cascade of acid stress resistance in *Escherichia coli*. BMC Microbiol. 10, 273 (2010). 21034467 10.1186/1471-2180-10-273 PMC2984483

[b44] Gaida S. M. *et al.* Synthetic tolerance: three noncoding small RNAs, DsrA, ArcZ and RprA, acting supra-additively against acid stress. Nucleic Acids Res. 41, 8726–8737 (2013). 23892399 10.1093/nar/gkt651 PMC3794604

[b45] Bak G., Han K., Kim D. & Lee Y. Roles of rpoS-activating small RNAs in pathways leading to acid resistance of *Escherichia coli*. Microbiologyopen 3, 15–28 (2014). 24319011 10.1002/mbo3.143 PMC3937726

[b46] Eguchi Y., Ishii E., Hata K. & Utsumi R. Regulation of acid resistance by connectors of two-component signal transduction systems in *Escherichia coli*. J. Bacteriol. 193, 1222–1228 (2011). 21193607 10.1128/JB.01124-10 PMC3067605

[b47] Kim D. *et al.* Comparative analysis of regulatory elements between *Escherichia coli* and *Klebsiella pneumoniae* by genome-wide transcription start site profiling. PLoS Genet. 8, e1002867 (2012). 22912590 10.1371/journal.pgen.1002867 PMC3415461

[b48] Burton N. A. *et al.* Novel aspects of the acid response network of *E. coli* K-12 are revealed by a study of transcriptional dynamics. J. Mol. Biol. 401, 726–742 (2010). 20603130 10.1016/j.jmb.2010.06.054

[b49] Mates A. K., Sayed A. K. & Foster J. W. Products of the *Escherichia coli* acid fitness island attenuate metabolite stress at extremely low pH and mediate a cell density-dependent acid resistance. J. Bacteriol. 189, 2759–2768 (2007). 17259322 10.1128/JB.01490-06 PMC1855797

[b50] Seputiene V. *et al.* Molecular characterization of the acid-inducible asr gene of *Escherichia coli* and its role in acid stress response. J. Bacteriol. 185, 2475–2484 (2003). 12670971 10.1128/JB.185.8.2475-2484.2003 PMC152617

[b51] Zhao B. & Houry W. A. Acid stress response in enteropathogenic gammaproteobacteria: an aptitude for survival. Biochem. Cell. Biol. 88, 301–314 (2010). 20453931 10.1139/o09-182

[b52] O'Brien E. J. *et al.* Genome-scale models of metabolism and gene expression extend and refine growth phenotype prediction. Mol. Syst. Biol. 9, 693 (2013). 24084808 10.1038/msb.2013.52 PMC3817402

[b53] Lu P. *et al.* L-glutamine provides acid resistance for *Escherichia coli* through enzymatic release of ammonia. Cell Res. 23, 635–644 (2013). 23337585 10.1038/cr.2013.13 PMC3641589

[b54] Richard H. & Foster J. W. *Escherichia coli* glutamate- and arginine-dependent acid resistance systems increase internal pH and reverse transmembrane potential. J. Bacteriol. 186, 6032–6041 (2004). 15342572 10.1128/JB.186.18.6032-6041.2004 PMC515135

[b55] Chattopadhyay M. K. & Tabor H. Polyamines are critical for the induction of the glutamate decarboxylase-dependent acid resistance system in *Escherichia coli*. J. Biol. Chem. 288, 33559–33570 (2013). 24097985 10.1074/jbc.M113.510552 PMC3837104

[b56] Bazzini A. A., Lee M. T. & Giraldez A. J. Ribosome profiling shows that miR-430 reduces translation before causing mRNA decay in zebrafish. Science 336, 233–237 (2012). 22422859 10.1126/science.1215704 PMC3547538

[b57] Ruder W. C., Lu T. & Collins J. J. Synthetic biology moving into the clinic. Science 333, 1248–1252 (2011). 21885773 10.1126/science.1206843

[b58] Warnecke T. & Gill R. T. Organic acid toxicity, tolerance, and production in *Escherichia coli* biorefining applications. Microb. Cell Fact. 4, 25 (2005). 16122392 10.1186/1475-2859-4-25 PMC1208944

[b59] Chang R. L. *et al.* Structural systems biology evaluation of metabolic thermotolerance in *Escherichia coli*. Science 340, 1220–1223 (2013). 23744946 10.1126/science.1234012 PMC3777776

[b60] Cho B. K., Knight E. M. & Palsson B. O. PCR-based tandem epitope tagging system for *Escherichia coli* genome engineering. Biotechniques 40, 67–72 (2006). 16454042 10.2144/000112039

[b61] Datta S., Costantino N. & Court D. L. A set of recombineering plasmids for gram-negative bacteria. Gene 379, 109–115 (2006). 16750601 10.1016/j.gene.2006.04.018

[b62] Cho B. K. *et al.* Genome-scale reconstruction of the Lrp regulatory network in *Escherichia coli*. Proc. Natl Acad. Sci. USA 105, 19462–19467 (2008). 19052235 10.1073/pnas.0807227105 PMC2614783

[b63] Rhee H. S. & Pugh B. F. ChIP-exo method for identifying genomic location of DNA-binding proteins with near-single-nucleotide accuracy. Curr. Protoc. Mol. Biol. Chapter 21, 21–24 (2012). 10.1002/0471142727.mb2124s100 PMC3813302 23026909

[b64] Seo S. W. *et al.* Deciphering Fur transcriptional regulatory network highlights its complex role beyond iron metabolism in *Escherichia coli*. Nat. Commun. 5, 4910 (2014). 25222563 10.1038/ncomms5910 PMC4167408

[b65] Langmead B., Trapnell C., Pop M. & Salzberg S. L. Ultrafast and memory-efficient alignment of short DNA sequences to the human genome. Genome. Biol. 10, R25 (2009). 19261174 10.1186/gb-2009-10-3-r25 PMC2690996

[b66] Wang L. *et al.* MACE: model based analysis of ChIP-exo. Nucleic Acids Res. 42, e156 (2014). 25249628 10.1093/nar/gku846 PMC4227761

[b67] Levin J. Z. *et al.* Comprehensive comparative analysis of strand-specific RNA sequencing methods. Nat. Methods 7, 709–715 (2010). 20711195 10.1038/nmeth.1491 PMC3005310

[b68] Trapnell C. *et al.* Transcript assembly and quantification by RNA-Seq reveals unannotated transcripts and isoform switching during cell differentiation. Nat. Biotechnol. 28, 511–515 (2010). 20436464 10.1038/nbt.1621 PMC3146043

[b69] Bailey T. L. *et al.* MEME SUITE: tools for motif discovery and searching. Nucleic Acids Res. 37, W202–W208 (2009). 19458158 10.1093/nar/gkp335 PMC2703892

[b70] Aziz R. K. *et al.* The RAST Server: rapid annotations using subsystems technology. BMC Genomics 9, 75 (2008). 18261238 10.1186/1471-2164-9-75 PMC2265698

