# Supplementary material for: Decoding genome-wide GadEWX-transcriptional regulatory networks reveals multifaceted cellular responses to acid stress in Escherichia coli
Source: Nat Commun. 2015 Aug 10;6:7970. doi: 10.1038/ncomms8970 (PMC4918353; doi:10.1038/ncomms8970)
Supplement: Supplementary Information — Supplementary Figures 1-7 and Supplementary Tables 1-5 [file ncomms8970-s1.pdf]

Supplementary Figures

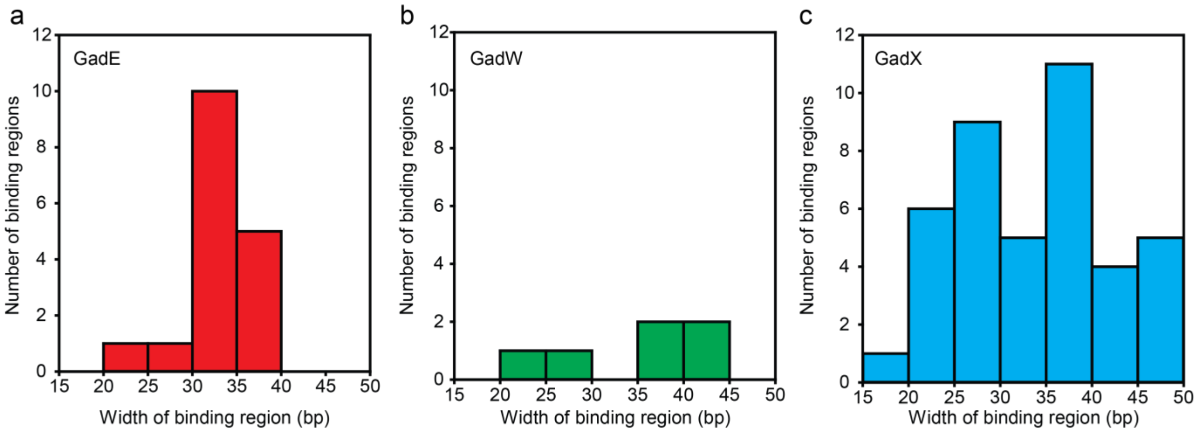

Supplementary Figure 1. Width distribution of ChIP-exo binding sites. The average binding widths for (**a**) GadE, (**b**) GadW, and (**c**) GadX are 29±3.6, 30±7.6, 28.7±8.0, respectively.

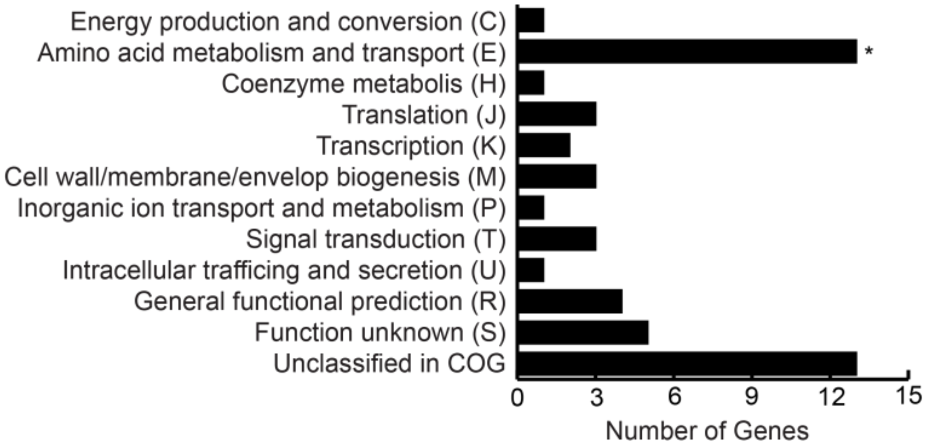

Supplementary Figure 2. Functional classification of genes that belong to GadEWX regulons. Clusters of Orthologous Groups (COG) categories were used to find the enriched functional groups. We performed one-tailed Fisher's exact test (Hypergeometric test), and *P-value* < *0.05* was considered significant. The asterisk indicates Hypergeometric *P-value* < 0.05.

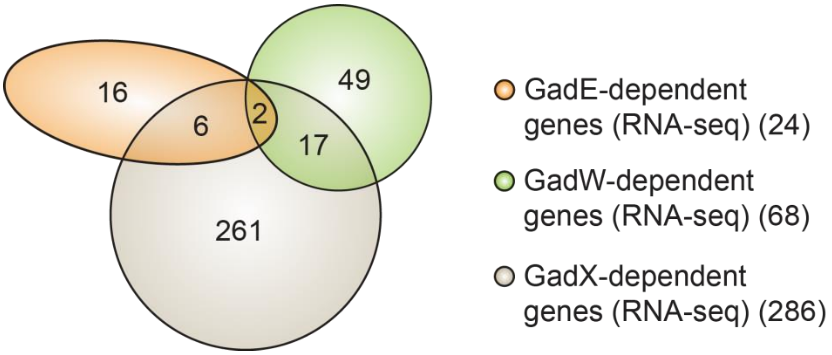

Supplementary Figure 3. Overlap among GadE-, GadW-, and GadX-dependent transcriptome under acidic stress. Transcription level was compared between wild-type and each deletion mutant to define dependent genes under acidic stress (pH 5.5).

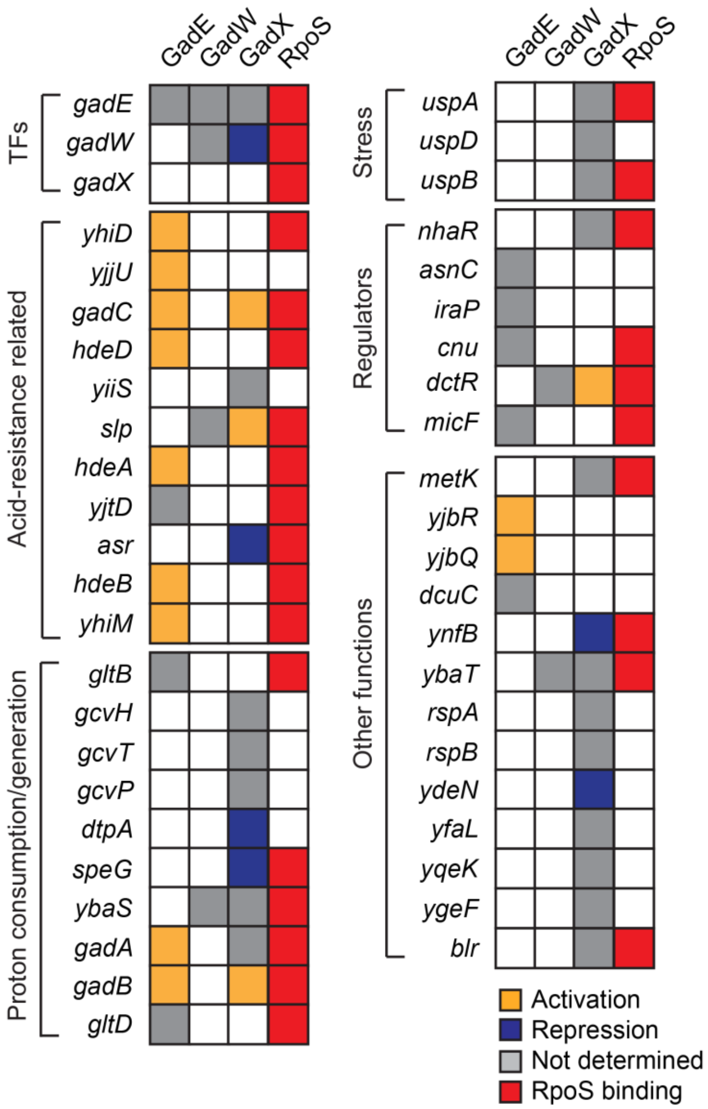

Supplementary Figure 4. Regulatory causation in GadE, GadW, and GadX regulons and RpoS interaction. Causal relationships between direct associations of transcription factors (GadE, GadW, and GadX) and the changes in transcript levels of genes. The binding events of RpoS under acid stress (pH 5.5) were also described.

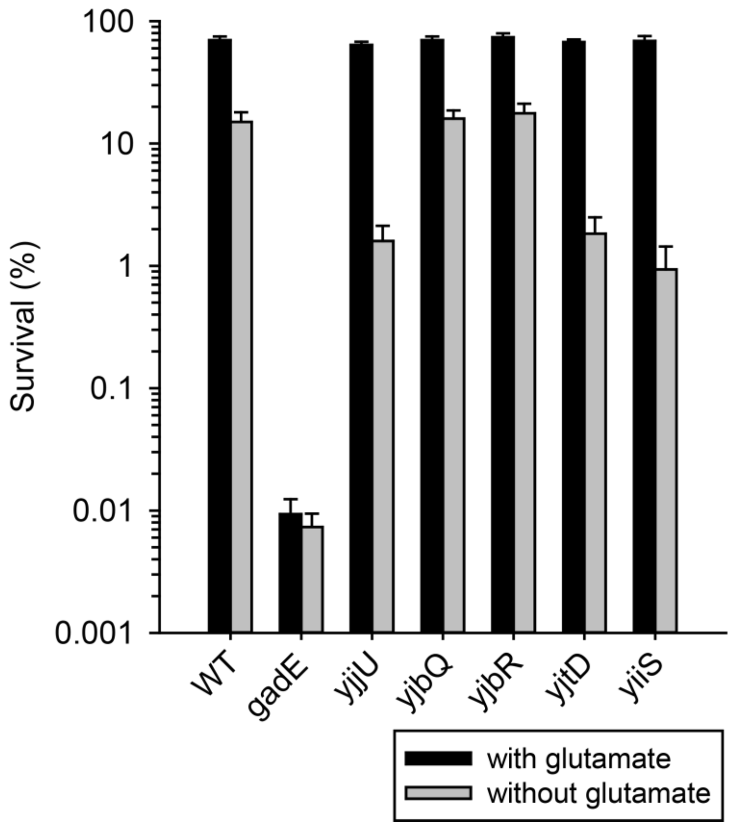

Supplementary Figure 5. Acid resistance assays. We tested acid resistance for knock-out strains of 5 different Y-genes whose functions are not yet described. They were not directly related to glutamate-dependent acid resistance such as GadE. However, three of them (*yjjU*, *yjtD*, and *yiiS*) were related to general acid resistance. The acid resistance assays were performed triplicates. The means and standard deviations (n=3) are depicted. Detailed procedures are described in the method section.

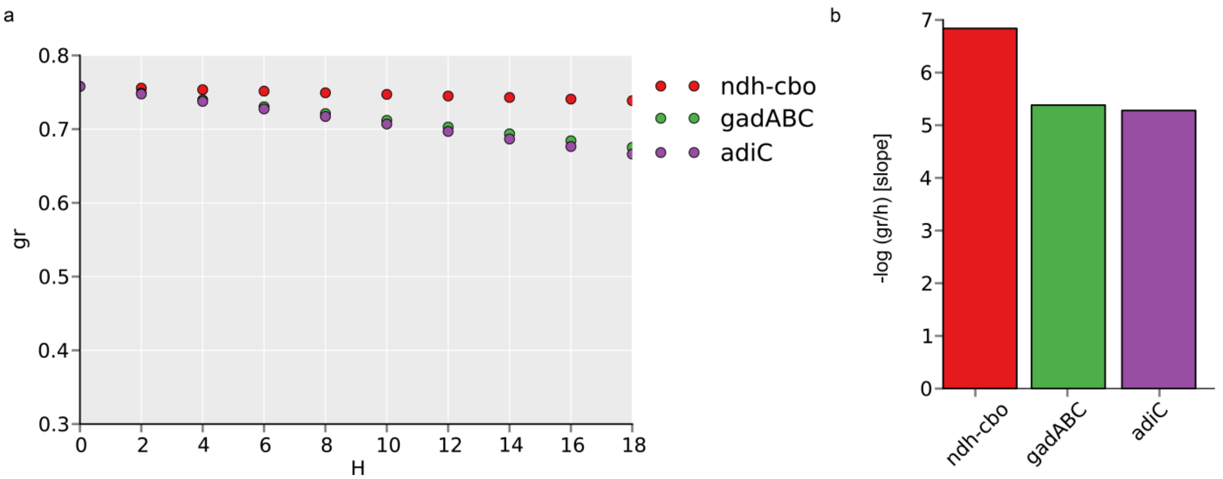

Supplementary Figure 6. ME-simulation results upon acid stress. From ME-model simulation, we predicted (**a**) changes of growth rate (gr) depending on the acid stress (H). (**b**) The slope (-log (gr/h)) was calculated to compare the efficiency of the relief of acid stress for each system. The ndh-cbo complexes were more efficient than other two amino acid decarbolyase-antiporter pairs.

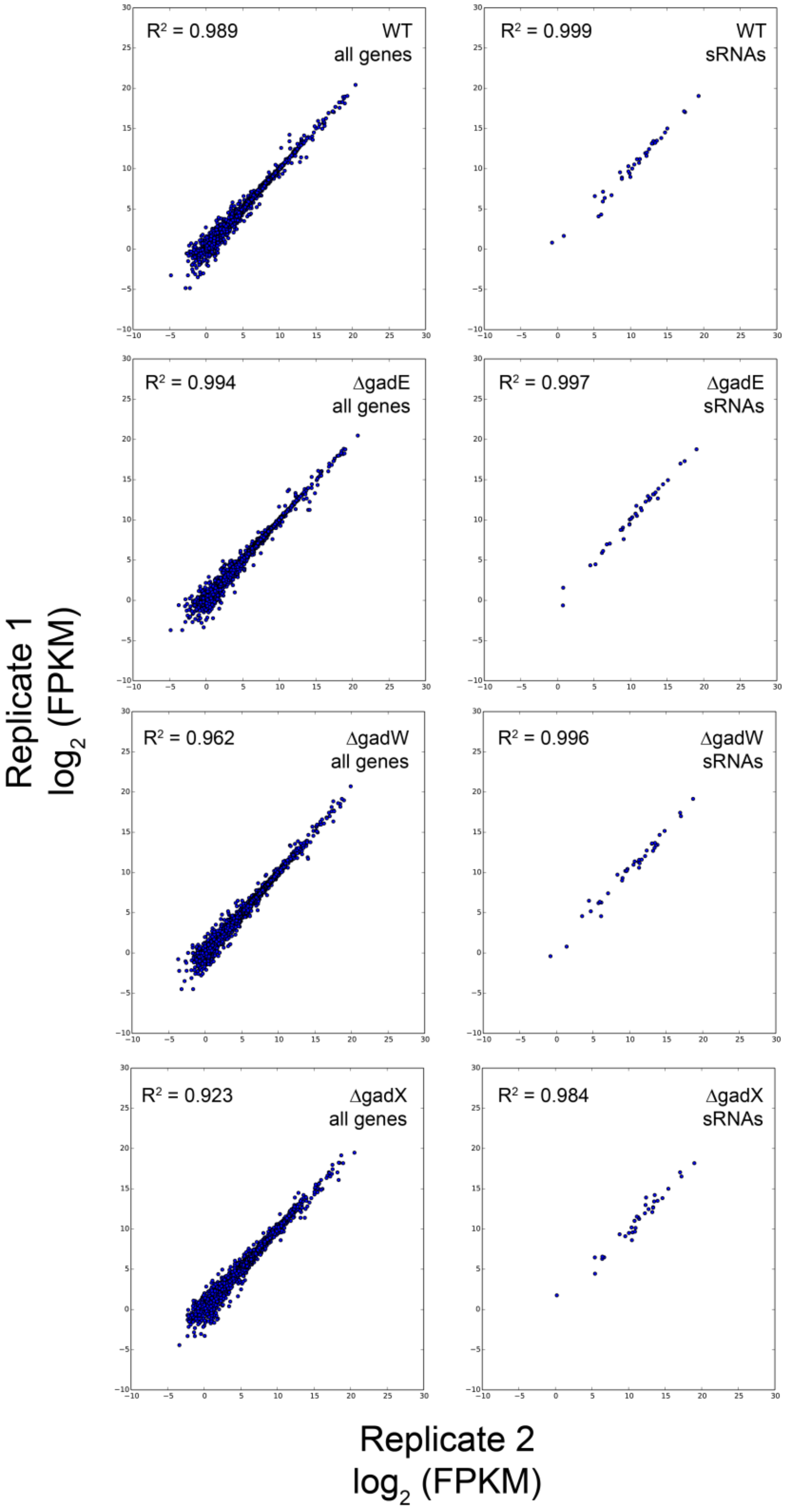

Supplementary Figure 7. Reproducibility of RNA-seq data. Log_2**(**FPKM) values of two biological replicates were compared for all genes and sRNAs for each strain.

Supplementary Tables

Supplementary Table 1. GadE-associated regions identified by ChIP-exo analysis.

Binding Sites	Transcription Unit	Peak	ChIP-exo Start	ChIP-exo End	Location	Causal relationship upon K/O	Regulatory Mode	Distance to TSS	S/N ratio	RpoS involved?[a]
1	*iraP*	P1	400316	400345	Upstream	N	-	-256.5	76.60	No
2	*dcuC*	P2	655216	655243	Upstream	N	-	76.5	1.43	No
3	*gadBC*	P3	1570144	1570175	Upstream	Y	Act	-62.5	2.78	Yes
4	*cnu*	P4	1702759	1702787	Upstream	N	-	-15	1.59	Yes
5	*micF*	P5	2311024	2311054	Upstream	N	-	-67	15.21	Yes
6	*yfjU*	P6	2769762	2769784	Downstream	N	-	-	1.15	-
7	*gltBD*	P7	3352240	3352258	Upstream	N	-	-284	1.20	Yes
8	*yhiM*	P8	3632675	3632700	Upstream	Y	Act	-78.5	1.53	Yes
9	*hdeAB-yhiD*	P9	3654789	3654820	Upstream	Y	Act	9.5	25.63	Yes
10	*hdeD*	P10	3654915	3654943	Upstream	Y	Act	-54	40.96	Yes
11	*gadE*	P11	3656193	3656227	Upstream	-	-	-55	13.48	Yes
12	*gadA*	P12	3665681	3665709	Upstream	Y	Act	-64	1.48	Yes
13	*asnC*	P13	3925018	3925047	Upstream	N	-	21.5	1.24	No
14	*yjbQR*	P14	4267976	4268006	Upstream	Y	Act	-40	1.01	No
15	*yjjU*	P15	4610316	4610345	Upstream	Y	Act	186.5	1.09	No
16	*yjtD*	P16	4638752	4638780	Upstream	N	-	-	2.49	Yes

[a] Red characters indicate novel RpoS bindings.

Supplementary Table 2. GadW-associated regions identified by ChIP-exo analysis.

Binding Sites	Transcription Unit	Peak	ChIP-exo Start	ChIP-exo End	Location	Causal relationship upon K/O	Regulatory Mode	Distance to TSS	S/N ratio	RpoS Involved? [a]
1	*ybaST*	P1	510790	510823	Upstream	N	-	11.5	13.36	Yes
2	*slp-dctR*	P2	3651968	3651984	Upstream	N	-	17	5.87	Yes
3	*gadE*	P3	3655735	3655770	Upstream	N	-	-70.5	3.99	Yes
		P4	3655819	3655843	Upstream	N	-	8	4.53	
4	*gadW*	P5	3662857	3662888	Upstream	N	-	-68.5	14.26	Yes
		P6	3662883	3662918	Upstream	N	-	-96.5	20.80	

[a] Red characters indicate novel RpoS bindings.

Supplementary Table 3. GadX-associated regions identified by ChIP-exo analysis.

Binding Sites	Transcription Unit	Peak	ChIP-exo Start	ChIP-exo End	Location	Causal relationship upon K/O	Regulatory Mode	Distance to TSS	S/N ratio	RpoS Involved?[a]
1	*nhaR*	P1	18012	18042	Upstream	N	-	2	102.70	Yes
2	*yagN*	P2	294529	294546	Internal	N	-	-	7.49	-
3	*ybaST*	P3	510790	510823	Upstream	N	-	11.5	417.36	Yes
4	*asnS*	P4	987539	987552	Internal	N	-	-	6.23	-
5	*gadBC*	P5	1570011	1570051	Upstream	Y	Act	66	50.10	Yes
		P6	1570261	1570301	Upstream			-184	42.20	
		P7	1570302	1570342	Upstream			-225	43.95	
		P8	1570345	1570385	Upstream			-268	41.97	
6	*ydeN*	P9	1580632	1580662	Upstream	Y	Rep	-69	8.32	No
		P10	1580799	1580824	Upstream			-233.5	13.67	
7	*rspAB*	P11	1653263	1653281	Upstream	N	-	395	7.82	No
8	*ynfB-speG*	P12	1653697	1653730	Upstream	Y	Rep	-94.5	3.97	Yes
9	*asr*	P13	1669352	1669375	Upstream	Y	Rep	473.5	1.83	Yes
10	*blr*	P14	1702520	1702552	Upstream	N	-	0	8.70	Yes
11	*dtpA*	P15	1710534	1710554	Upstream	Y	Rep	-151	2.54	No
12	*yfaL*	P16	2342146	2342173	Upstream	N	-	-	7.84	No
		P17	2342169	2342207	Upstream			-	8.54	
13	*yqeK/ygeF*	P18	2988594	2988613	Upstream	N	-	-	23.57	No
14	*ygeH*	P19	2990931	2990949	Internal	N	-	-	7.45	-
15	*gcvTHP*	P20	3048878	3048906	Upstream	N	-	-98	8.66	No
		P21	3048982	3049002	Upstream			-198	10.94	
16	*metK*	P22	3084478	3084504	Upstream	N	-	-100	19.66	Yes
		P23	3084574	3084607	Upstream			-0.5	45.18	
		P24	3084677	3084707	Upstream			101	26.52	
17	*uspA/uspB*	P25	3637850	3637894	Upstream	N	-	-135/-3	4.82	Yes
18	*yhiS_1*	P26	3649418	3649455	Internal	N	-	-	32.10	-

		P27	3651832	3651858	Upstream			-114	145.28	
		P28	3651850	3651872	Upstream			-98	178.00	
19	*slp-dctR*	P29	3651863	3651894	Upstream	Y	Act	-80.5	151.59	Yes
		P30	3651946	3651966	Upstream			-3	179.26	
		P31	3651968	3651984	Upstream			17	192.68	
		P32	3655735	3655770	Upstream			-70.5	66.35	
20	*gadE*	P33	3655754	3655785	Upstream	N	-	-53.5	54.04	Yes
		P34	3655775	3655798	Upstream			-36.5	40.39	
		P35	3655811	3655842	Upstream			3.5	79.18	
		P36	3662816	3662836	Upstream			-22	118.48	
21	*gadW*	P37	3662857	3662888	Upstream	Y	Rep	-68.5	174.99	Yes
		P38	3662883	3662918	Upstream			-96.5	337.74	
22	*gadA*	P39	3665727	3665747	Upstream	N	-	-106	11.37	Yes
		P40	3665749	3665768	Upstream			-127.5	14.58	
23	*yiiS-uspD*	P41	4110801	4110821	Upstream	N	-	71	43.24	No

[a] Red characters indicate novel RpoS bindings.

Supplementary Table 4. Confirmation of previously characterized GadEWX-binding sites

Number	Transcription Unit	Decision
	**GadE**	
1	*gadBC*	Yes
2	*rcsA*	No
3	*gltBD*	Yes
4	*hdeAB-yhiD*	Yes
5	*hdeD*	Yes
6	*gadE*	Yes
7	*gadX*	No
	**GadW**	
1	*gadBC*	No
2	*hdeAB-yhiD*	No
3	*gadA*	No
	**GadX**	
1	*gadBC*	Yes
2	*slp-dctR*	Yes
3	*hdeAB-yhiD*	No
4	*hdeD*	No
5	*gadA*	Yes
6	*btuB*	No
	Total	8/16 (50%)

Supplementary Table 5. Other transcription factors known to regulate genes in the GadEWX regulons (45 genes).

TFs	# of binding sites	Target genes
H-NS	9	*yhiD/gadE/slp/gadA/hdeD/hdeB/hdeA/yhiM/dctR*
FliZ	6	*yhiD/gadE/gadB/gadC/hdeB/hdeA*
CRP	6	*gcvP/gadE/gcvT/gcvH/gltB/gltD*
Lrp	6	*gcvP/gcvT/gcvH/micF/gltB/gltD*
AdiY	5	*gadC/gltB/gltD/gadB/gadA*
PhoP	4	*yhiD/hdeD/hdeB/hdeA*
MarA	4	*yhiD/micF/hdeB/hdeA*
IHF	3	*micF/gltB/gltD*
GcvA	3	*gcvH/gcvP/gcvT*
OmpR	2	*micF/dtpA*
HdfR	2	*gltB/gltD*
ArgR	2	*gltB/gltD*
FadR	1	*uspA*
Rob	1	*micF*
SoxS	1	*micF*
NhaR	1	*nhaR*
ArcA	1	*gadE*
EvgA	1	*gadE*
RcsB	1	*gadA*
RcdA	1	*asr*
RstA	1	*asr*
YdeO	1	*gadE*
